# Determination of mean corpuscular haemoglobin cut-off point for differentiating alpha plus and alpha zero thalassaemia in thalassaemia screening

**DOI:** 10.1007/s00277-026-06868-7

**Published:** 2026-02-28

**Authors:** Ezalia Esa, Lailatul Hadziyah Mohd Pauzy, Hafizah Hashim, Azian Naila Md Nor, Ermi Neiza Mohd Sahid, Yuslina Mat Yusoff, Nur Aisyah Aziz, Faidatul Syazlin Abdul Hamid, Syahzuwan Hassan, Wahyuni Sofia Foo Mohammad Salleh, Ezzanie Suffya Zulkefli, Norafiza Mohd Yasin

**Affiliations:** 1 Virology Unit, Infectious Disease Research Centre, Institute for Medical Research, National Institutes of Health, Shah Alam, Selangor 40170 Malaysia; 2 Haematology Unit, Cancer Research Centre, Institute for Medical Research, National Institutes of Health, Shah Alam, Selangor Malaysia; 3 Department of Pathology, Hospital Sultanah Bahiyah, Alor Setar, Kedah Malaysia

**Keywords:** Deletional alpha thalassaemia, Alpha zero thalassaemia carriers, Alpha plus thalassaemia carriers, MCH cut-off, Malaysia thalassaemia diagnosis code

## Abstract

This study assesses haematological parameters as screening tools for identifying α zero (α⁰) thalassaemia candidates for DNA analysis. The Malaysia Thalassaemia Diagnosis Code (MTDC) uses mean corpuscular haemoglobin (MCH) < 25 pg to screen for suspected α⁰ thalassaemia carriers. Six haematological parameters from 304 cases genotyped by Gap-Polymerase Chain Reaction or Multiplex Ligation-dependent Probe Amplification were analyzed. Among the cases, 160 individuals (52.6%) were α^+^ thalassaemia silent carrier (-α/αα), comprising heterozygotes for -α^3.7^, -α^4.2^, –(α)^20.5^ and -α^2.4^. Twelve individuals (3.9%) were α^+^ thalassaemia carrier (-α/-α), including homozygous -α^3.7^ and compound heterozygous -α^3.7^ and -α^4.2^. Meanwhile, 111 individuals (36.4%) were α^0^ thalassaemia carrier (--/αα) consisting of heterozygotes for --^SEA^, --^GB^, --^FIL^, --^AW^, and –^THAI^ deletions. Deletional Hb H disease (--/-α) was observed in individuals with compound genotypes such as -α^3.7^/--^SEA^, -α^3.7^/--^GB^, -α^3.7^/--^AW^, -α^4.2^/--^SEA^ and -α^2.4^/--^SEA^. All parameters correlated with the number of α-globin gene deletions, with mean corpuscular volume (MCV) (r^2^ = 0.73) and MCH (r^2^ = 0.77) showing the strongest associations. Gender-specific MCH cut-offs of < 22.65 pg for males and < 22.25 pg for females demonstrated excellent diagnostic performance, with higher sensitivity and specificity in females (90.3% and 93.9%) than in males (89.5% and 88.9%). The findings indicate that the currently used MCH cut-off of 25 pg may be higher than optimal for identifying α⁰ thalassaemia carriers. A lower MCH threshold improves detection performance and may support refinement of the MTDC screening criteria to better prioritize high-risk individuals while reducing unnecessary DNA testing among α⁺ thalassaemia cases.

## Introduction

Alpha (α) thalassaemia is a common genetic disorder caused by deletions in the α-globin genes (*HBA1* and *HBA2*), with disease severity determined by the number of affected genes. Clinical phenotypes range from silent carriers with minimal haematological changes to severe forms such as Hb H disease and Hb Bart hydrops foetalis syndrome [1–4]. In Malaysia, the -α³·⁷ deletion is the most prevalent α⁺ thalassaemia among Malays, Sabahans, and Malaysian Indians, whereas α⁰ thalassaemia, particularly the --^SEA^ deletion, predominates among Malaysian Chinese [5–8]. Individuals from all ethnic groups who carry α⁰ thalassaemia are at risk of having offspring with Hb H disease or Hb Bart hydrops foetalis if their partners also carry α-globin gene deletion(s).

Screening for α thalassaemia typically involves a full blood count (FBC), capillary electrophoresis (CE), and/or high-performance liquid chromatography (HPLC), while confirmation of the genotype requires DNA analysis. To ensure consistent interpretation of these results, the National Thalassaemia Committee of Malaysia developed the Malaysia Thalassaemia Diagnosis Code (MTDC) to standardise the reporting of haemoglobin (Hb) analysis and DNA analysis results. In practice, laboratories classify individuals using MTDC codes based on Hb analysis results, which then guide the need for confirmatory DNA testing. Once DNA analysis is performed, cases are recoded according to the genotype identified. This coding scheme ensures consistent communication of findings across hospitals, clinics, and screening programmes.

For example, the D13 code is assigned to cases with MCH < 25 pg, in which double α-globin gene deletions are suspected and DNA confirmation is required. In contrast, the D16 code applies to MCH values of 25–27 pg, which are typically indicative of α⁺ thalassaemia and do not necessarily require DNA analysis. Although current BSH guidelines recommend MCH < 27 pg to trigger DNA testing for α⁰ thalassaemia, recent studies suggest that lower MCH thresholds may improve differentiation between α⁺ and α⁰ thalassaemia carriers [1, 8, 9].

This study aimed to describe haematological parameters for deletional α thalassaemia, focusing on evaluating MCH cut-offs to enhance screening algorithms for distinguishing α⁺ and α⁰ thalassaemia carriers in the Malaysian population.

## Materials and methods

### Study subjects

The Institute for Medical Research (IMR) functions as a secondary referral center for molecular diagnosis of α thalassaemia in Malaysia, receiving complex cases for further testing due to limitations in the techniques employed by primary molecular laboratories. Therefore, most of the cases selected for this study were derived from parental samples of complicated index cases and further testing for α thalassaemia. The samples were peripheral blood collected in EDTA tubes and transported under chilled conditions. Only samples with no evidence of clotting, haemolysis, insufficient volume or improper labelling were accepted. DNA extraction was performed using QIA symphony SP (Qiagen, GmbH). The concentration and quality of the extracted DNA were measured using a NanoDrop 1000 spectrophotometer (Thermo Fisher Scientific Inc). Samples with A260/280 ratios outside the range of 1.7–2.0 or with insufficient DNA concentration were excluded.

In this observational study, we retrospectively selected all confirmed cases diagnosed with deletional α thalassaemia by DNA analysis between January 2017 and December 2021. Only cases with complete clinical information and full haematological parameters were included. Individuals below 13 years of age, cases of α thalassaemia co-inherited with β thalassaemia or other haemoglobinopathies, compound deletional α thalassaemia with an α variant mutation, and uncharacterized deletions were excluded.

## FBC and hb analysis

Results of initial blood work, including FBC and hb analysis, were recorded. These screening tests were conducted by the referring hospital using automated Haematology analyzers, Sebia CE system (Sebia, France), and/or Bio-Rad Variant or Variant II HPLC systems (Bio-Rad Laboratories, Hercules, CA, USA). Normal reference ranges for complete blood counts were obtained from published data [10], whereas reference intervals for Hb A₂ were determined locally (CE: 2.0–3.2%; HPLC: 2.2–3.4%). Presumptive diagnoses derived from these screening tests, along with their corresponding diagnosis codes, were also recorded. The codes used were adopted from the MTDC for Hb analysis reporting (Table 1). As this study focuses on haematological parameters, only the Hb analysis codes are presented; the MTDC DNA analysis codes, although used in practice, are not shown here.

**Table 1:** Malaysia thalassaemia diagnosis code (MTDC) used for hb analysis reporting

**α thalassaemia**		**β thalassaemia**		
Hb H disease	A1	β thalassaemia trait	B1	
Hb Constant Spring	A2	β thalassaemia trait with iron deficiency (if iron status known)	B2	
Hb H disease: Hb H-Hb Constant Spring	A3	β thalassaemia intermedia	B3	
Hb H disease: Hb H-Hb Q	A4	β thalassaemia major	B4	
Hb Barts Hydrops Foetalis	A5	β thalassaemia trait with Hb variant	B5	
Hb H disease with other Hb variant	A6	β thalassaemia trait with suspected alpha thalassaemia/iron deficiency	B6	
Hb Constant Spring Homozygous	A7	β thalassaemia trait with suspected triplication alpha	B7	
**Haemoglobinopathies/Thalassaemia Haemoglobinopathies**				
Hb E Heterozygous/Hb E trait	C1	Hb S trait	C7	
Hb E Homozygous/Hb E disease	C2	Hb S with iron deficiency (if iron status known)	C8	
Hb E β thalassaemia	C3	Hb S with suspected α thalassaemia/iron deficiency	C9	
Hb E with iron def (if iron status known)	C4	Hb S Homozygous	C10	
Hb E with suspected α thalassaemia/Iron def	C5	Hb S β interaction	C11	
Hb E with coinheritance Hb variant (e.g. Hb Malay, D, J, C, S etc.)	C6	Hb Q Thailand	C12	
		Hb E Beta with suspected α thalassaemia/iron deficiency	C13	
**Haemoglobin variant (Pending Molecular Confirmation)**				
Hb C	D1	α thalassaemia (double gene deletion). DNA analysis for α globin gene required (MCH < 25pg)	D13	
Hb D	D2			
Hb J	D4	Borderline Hb A2 β thalassaemia trait (CE: A2 ≥ 3.3%, HPLC: ≥ 3.4%). DNA analysis for β globin gene is required	D14	
Hb Lepore	D7			
Hb O Indonesia	D10	Other Hb variants	D15	
Hereditary Persistent Foetal Haemoglobin (HPFH) or Delta Beta Thalassaemia (Hb F level ≥ 5%)	D12	α thalassaemia cannot be excluded (MCH 25.0–26.0.9pg) and MCV < 80fL, Hb normal	D16	
**Others**				
Iron deficiency anaemia (if iron status known)	E1	Miscellaneous (e.g. Post Transplant, Juvenile Myelomonocytic Leukaemia)	E5	
Suspected iron deficiency anaemia	E2	No abnormal result detected	N	
High F due to acquired/physiology (e.g. pregnancy, age, sepsis)	E3	Rejected sample (need to repeat e.g. clotted, leaking, insufficient)	R1	
Inconclusive (e.g. recent transfusion, severe anaemia, storage changes)	E4	Rejected sample (redundant request)	R2	
		Rejected sample (not indicated)	R3	

### DNA analysis

Whole blood specimens were collected in ethylenediaminetetraacetic acid (K_3_EDTA) tubes and transported at a chilled temperature. Genomic DNA extraction was performed using the standard protocol of Qiagen Inc. (Valencia, CA, USA). A Multiplex Gap-Polymerase Chain Reaction (PCR) screening panel, consisting of seven deletions including -α^3.7^, -α^4.2^, -(α)^20.5^, --^SEA^, --^FIL^, --^THAI^, and --^MED^ deletions, was employed to detect the most common deletion in the α-globin gene in our region [11]. The non-deletional α thalassaemia, such as codon 142/termination codon (TAA > CAA) Hb Constant Spring, codon 125 (CTG > CCG) Hb Quong Sze, codon 59 (GGC > GAC) Hb Adana, initiation codon (ATG > A-G), codon 30 (-GAG), codon 35 (TCC > CCC) Hb Evora, was excluded using an Amplification Refractory Mutation System (ARMS) analysis established previously [12]. Further analysis was done using Multiplex Ligation-dependent Probe Amplification (MLPA) (Salsa MLPA P140 HBA; MRC-Holland, Amsterdam, the Netherlands) to detect rarer deletions according to the manufacturer’s instructions. MLPA data were analysed using Coffalyser.Net software, with dosage ratios interpreted according to the standard thresholds: a ratio of ~ 1 indicated a normal copy number, ~ 0.5 indicated a heterozygous deletion, and ~ 0 indicated a homozygous deletion. Rare deletions such as -α^2.4^, --^GB^, --^AW^ were initially flagged based on probe-specific signal patterns and further confirmed by cross-referencing with published primers in Gap-PCR assays [13–16].

Some cases with a suspected diagnosis of Hb A_2_ β thalassaemia (coded as D14) underwent Sanger sequencing and MLPA (Salsa MLPA P102 HBB; MRC-Holland, Amsterdam, the Netherlands) testing on the β-globin gene to rule out the presence of β-globin gene mutations and/or deletions.

### Statistical analysis

Cases were classified according to the number of deleted α-globin genes: individuals with a single gene deletion were categorized as α⁺ thalassaemia silent carriers; those with two gene deletions were classified as α⁺ or α⁰ thalassaemia carriers, depending on the deletion type; and those with three gene deletions were categorized as deletional Hb H disease.

Sample size calculation was based on mean corpuscular haemoglobin (MCH), selected as the primary haematological parameter for screening and differentiating α⁺ and α⁰ thalassaemia carriers. Although several haematological indices such as Hb, Mean Corpuscular Volume (MCV), and Mean Corpuscular Hemoglobin Concentration (MCHC) have been reported to differ significantly between genotypic groups, MCH was chosen because it directly reflects hypochromia and globin chain imbalance, key pathophysiological features of α-thalassaemia [17]. In addition, MCH is less influenced by physiological and analytical variability than Hb and MCV, and it is routinely available and highly reproducible in complete blood count analysis, making it suitable for screening purposes [9, 18, 22].

Because some haematological parameters (e.g., red blood cell [RBC], Hb, and MCHC) differ biologically between males and females [10], all analyses including red cell distribution width (RDW) and Hb A_2_ were stratified by sex. Independent samples t-tests were used to compare these parameters between the combined α⁺ group (α⁺ silent + α⁺ carriers) and α⁰ thalassaemia carriers, with p-values < 0.05 considered statistically significant. Based on previously published data (combined α⁺ mean ~ 23.0 pg, SD ~ 3 pg; α⁰ mean ~ 21.2 pg, SD ~ 2 pg) [5], the expected difference in MCH would require ~ 37 participants per group to achieve 80% power at a two-sided significance level of 0.05.

Receiver operating characteristic (ROC) curves were also constructed to evaluate these haematological parameters in differentiating α⁺ and α⁰ thalassaemia carriers. The ROC-based sample size was calculated prospectively using an estimated AUC of 0.75–0.8 from a previous study [5], indicating a minimum of 25 participants per group for 80% power and a two-sided significance level of 0.05.

## Results

### Deletional α thalassaemia

Of the 304 α thalassaemia cases, 192 (63.2%) were females and 112 (36.8%) were males. The higher number of females may be due to antenatal screening and anaemia investigations in Form Four students. The median age was 29 years (range: 13–63), with the majority (*n* = 44, 14.5%) being Form Four students from the National Thalassaemia Screening Programme. Most cases in the α^+^ thalassaemia silent carrier group (*n* = 160, 52.6%), followed by α⁰ thalassaemia carrier (*n* = 111, 36.4%), deletional Hb H disease (*n* = 21, 6.9%), and α^+^ thalassaemia carrier (*n* = 12, 3.9%). The -α^3.7^ was predominantly found in Malays, with 81.5% (119/146) of heterozygous and 55.6% (5/9) of homozygous cases. The heterozygous --^SEA^ deletion was common in Malays (43/71, 60.6%) and Chinese (22/71, 31.0%). Compound heterozygosity for --^SEA^ and -α^3.7^ deletions was also mainly seen in Malays (13/15, 86.7%). Table 2 summarizes the specific genotype of deletions and their classifications. The distribution of the genotype across the different race are shown in Table 3 and the haematological parameters for each genotype of deletional α thalassaemia were summarized in Table 4.

**Table 2:** Frequency of deletional α thalassaemia

Genotypes of deletional α thalassaemia & HGVS nomenclature	Number of cases, *n* (%)
One gene deletion, (-α/αα), α^+^ Thalassaemia Silent Carrier	160 (52.6)
Heterozygous -α^3.7^ deletion, NG_000006.1:g.[34164_37967del]; [34164_37967=]	146 (48.0)
Heterozygous -α^4.2^ deletion, NG_000006.1:g.[30682_34939del]; [30682_34939=]	12 (3.9)
Heterozygous –(α)^20.5^ deletion, NG_000006.1:g.[15164_37864del]; [15164_37864=]	1(0.3)
Heterozygous -α^2.4^ deletion^†^, NG_000006.1:g.[36854_39245del]; [36854_39245=]	1(0.3)
Two genes deletion, (-α/-α), α^+^ Thalassaemia Carrier	12 (3.9)
Homozygous -α^3.7^ deletion, NG_000006.1:g.[34164_37967del]; [34164_37967del]	9 (3.0)
Compound heterozygous -α^3.7^ and -α^4.2^ deletions, NG_000006.1:g.[34164_37967del]; [30682_34939del]	3 (1.0)
Two genes deletion, (--/αα), α^0^ Thalassaemia Carrier	111 (36.4)
Heterozygous --^SEA^ deletion, NG_000006.1:g.[26264_45564del]; [26264_45564=]	71 (23.4)
Heterozygous --^GB^ deletion^†^, NG_000006.1:g.[22766_39536del]; [22766_39536=]	27 (8.9)
Heterozygous state of --^FIL^ deletion, NG_000006.1:g.[12483_43158del]; [12483_43158=]	5 (1.6)
Heterozygous state of --^AW^ deletion^†^, NG_000006.1:g.[32143_40317del]; [32143_40317=]	6 (2.0)
Heterozygous --^THAI^ deletion, NG_000006.1:g.[10726_44715del]; [10726_44715=]	2 (0.7)
Three genes deletion, (--/-α), Deletional Hb H Disease	21 (6.9)
Compound heterozygous -α^3.7^ and --^SEA^ deletions, NG_000006.1:g.[34164_37967del]; [26264_45564del]	15 (4.9)
Compound heterozygous -α^3.7^ and --^GB^ deletions, NG_000006.1:g.[34164_37967del]; [22766_39536del]	3 (1.0)
Compound heterozygous -α^3.7^ and --^AW^ deletions, NG_000006.1:g.[34164_37967del]; [32143_40317del]	1 (0.3)
Compound heterozygous -α^4.2^ and --^SEA^ deletions, NG_000006.1:g.[30682_34939del]; [26264_45564del]	1 (0.3)
Compound heterozygous -α^2.4^ and --^SEA^ deletions, NG_000006.1:g.[36854_39245del]; [26264_45564del]	1 (0.3)
**Grand Total**	**304**

The frequency of each deletional α thalassaemia is expressed as the specific number and percentage of the total sample size

† The rarer deletions are presented alongside their HGVS nomenclature

*n* number of cases

**Table 3:** Distribution of deletional α thalassaemia across racial groups

Genotype		α^+^ Thal Silent Carrier (-α/αα)	α^+^ Thal Carrier (-α/-α)	α^0^ Thal Carrier (--/αα)	Del Hb H Disease (--/-α)
Race	Gender	Male: *n* = 63 Female: *n* = 97	Male: *n* = 3 Female: *n* = 9	Male: *n* = 39 Female: *n* = 72	Male: *n* = 7 Female: *n* = 14
Malay	Male	51	2	26	6
	Female	80	6	51	12
Chinese	Male	3		12	
	Female	2		11	2
Sabah Indigenous	Male	5	1		
	Female	5	2	4	
Sarawak Indigenous	Male	1		1	
	Female	4		1	
Indian	Male				
	Female	2	1		
Others	Male	1			1
	Female	1		3	
Unknown	Male	2			
	Female	3		2	

*Thal* thalassaemia, *Del* deletion

**Table 4:** Haematologic parameters of the different genotypes of deletional α thalassaemia

Genotype		α^+^ Thal Silent Carrier (-α/αα)	α^+^ Thal Carrier (-α/-α)	α^0^ Thal Carrier (--/αα)	Del Hb H Disease (--/-α)
Parameters (unit)	Gender				
RBC (x 10^12^/L)	Male	5.74 (± 0.64), *n* = 63	6.12 (± 0.11), *n* = 3	6.66 (± 0.59), *n* = 39	5.51 (± 0.80), *n* = 7
	Female	4.86 (± 0.46), *n* = 97	4.89 (± 0.51), *n* = 9	5.58 (± 0.78), *n* = 72	4.84 (± 0.90), *n* = 14
Hb (g/dL)	Male	14.70 (1.49), *n* = 63	13.67 (± 0.40), *n* = 3	13.98 (± 1.04), *n* = 39	9.83 (± 1.15), *n* = 7
	Female	11.95 (± 1.31), *n* = 97	11.01 (± 1.11), *n* = 9	11.31 (± 1.16), *n* = 72	8.59 (± 1.16), *n* = 14
MCV (fL)	Male	79.02 (± 5.67), *n* = 63	71.83 (± 1.76), *n* = 3	67.33 (± 4.14), *n* = 39	64.57 (± 5.61), *n* = 7
	Female	76.88 (± 5.60), *n* = 97	71.57 (± 4.90), *n* = 9	66.29 (± 4.04), *n* = 72	60.30 (± 6.27), *n* = 14
MCH (pg)	Male	25.69 (± 2.22), *n* = 63	22.33 (± 0.92), *n* = 3	21.02 (± 1.14), *n* = 39	18.20 (± 0.86), *n* = 7
	Female	24.65 (± 2.41), *n* = 97	22.56 (± 1.57), *n* = 9	20.45 (± 1.77), *n* = 72	17.98 (± 1.92), *n* = 14
MCHC (g/L)	Male	32.47 (± 1.38), *n* = 63	31.10 (± 0.56), *n* = 3	31.27 (± 1.22), *n* = 39	28.31 (± 2.07), *n* = 7
	Female	32.04 (± 1.74), *n* = 97	31.51 (± 0.90), *n* = 9	30.83 (± 2.21), *n* = 72	29.85 (± 1.50), *n* = 14
RDW (%)	Male	14.42 (± 3.04), *n* = 55	13.93 (± 1.16), *n* = 3	16.21 (± 2.11), *n* = 37	26.16 (± 1.87), *n* = 7
	Female	14.56 (± 2.12), *n* = 89	15.34 (± 1.45), *n* = 9	16.36 (± 2.45), *n* = 67	23.94 (± 4.01), *n* = 13
Hb A_2_ (%)	Male	2.91 (± 0.44), *n* = 62	2.40 (± 0.56), *n* = 3	2.41 (± 0.28), *n* = 38	1.61 (± 0.82), *n* = 7
	Female	2.83 (± 0.54), *n* = 92	2.69 (± 0.32), *n* = 9	2.51 (± 0.45), *n* = 71	1.39 (± 0.50), *n* = 14

Data represent mean ± SD (standard deviation). Most groups met the minimum sample size (15 per group) except the α^+^ Thal Carrier group

### Mapping of DNA analysis results with hb analysis codes

The distribution of Hb analysis codes for the deletional α thalassaemia cases in this study are presented in Table 5. In the α^+^ thalassaemia silent carrier group, most cases were coded as D14 (*n* = 50, 31.2%, Hb A_2_ mean: 3.5%) and D16 (*n* = 50, 31.1%). The α⁰ thalassaemia carrier group and the α^+^ thalassaemia carrier group were mainly coded as D13, with 92.8% (*n* = 103) and 91.7% (*n* = 11), respectively. A case coded as A1 in α⁰ thalassaemia carrier was genotyped as a heterozygous state of --^AW^ deletion. For the deletional Hb H disease group, the majority (*n* = 17, 81.0%) were coded as A1 (defined as Hb H disease in MTDC), which concordance with their genotype.

**Table 5:** The hb analysis codes obtained from the records for study subjects

Genotype		α^+^ Thal Silent Carrier (-α/αα)	α^+^ Thal Carrier (-α/-α)	α^0^ Thal Carrier (--/αα)	Del Hb H Disease (--/-α)
MTDC code	Gender	Male: *n* = 63 Female: *n* = 97	Male: *n* = 3 Female: *n* = 9	Male: *n* = 39 Female: *n* = 72	Male: *n* = 7 Female: *n* = 14
N	Male	10 (6.3%)	-	-	-
	Female	9 (5.6%)	-	-	-
A1	Male	-	-	-	6 (28.6%)
	Female	-	-	1 (0.9%)	11 (52.4%)
D13	Male	10 (6.3%)	3 (25.0%)	39 (35.1%)	1 (4.8%)
	Female	22 (13.8%)	8 (66.7%)	64 (57.7%)	2 (9.5%)
D14	Male	17 (10.6%)	-	-	-
	Female	33 (20.6%)	1 (8.3%)	4 (3.6%)	-
D16	Male	25 (15.6%)	-	-	-
	Female	25 (15.6%)		-	-
Other codes	Male	E2: 1 (0.6%)	-	-	-
	Female	E2: 4 (2.5%), A2: 1 (0.6%), B6: 1 (0.6%), D2: 1(0.6%), D15: 1(0.6%)	-	E2: 3 (2.7%)	D15: 1 (4.8%)

The frequency of each deletion is expressed as the specific number and percentage from the total number of cases for each genotype

The definition for each MTDC code is shown in Table 1

### Correlation between haematological parameters and number of α-globin genes

Pearson correlation analysis found that all haematological parameters were correlated with the number of α-globin genes deleted (*p* < 0.01) with MCV and MCH showing the strongest correlations at r^2^ = 0.73 and r^2^ = 0.77 each, followed by RDW with r^2^=−0.54.

### Alpha plus thalassaemia versus alpha zero thalassaemia

No significant differences were observed in the mean haematological parameters between α⁺ thalassaemia silent carriers (-α/αα) and α⁺ thalassaemia carriers (-α/-α). Therefore, these two groups were combined into a single α⁺ thalassaemia group for comparison with the α⁰ thalassaemia carrier group (--/αα). Given their comparable haematological profiles, this pooling was considered appropriate and facilitated clearer discrimination of the α⁰ thalassaemia carrier group, while also improving statistical power for the screening analysis. All the haematological parameters were significantly different between these two groups (Table 6).

**Table 6:** Comparison of haematologic parameters of subjects with α^+^ and α⁰ thalassaemia Carrrier

Genotype		α^+^ Thalassaemia (-α/αα) and (-α/-α)	α^0^ Thalassaemia Carrier (--/αα)	P-value
Parameters (unit)	Gender			
RBC (x 10^12^/L)	Male	5.75 (± 0.63), *n* = 66	6.66 (± 0.59), *n* = 39	< 0.000
	Female	4.87 (± 0.46), *n* = 106	5.58 (± 0.78), *n* = 72	< 0.000
Hb (g/dL)	Male	14.66 (± 1.47), *n* = 66	13.98 (± 1.04), *n* = 39	0.013
	Female	11.87 (± 1.31), *n* = 106	11.31 (± 1.16), *n* = 72	0.04
MCV (fL)	Male	78.70 (± 5.74), *n* = 66	67.33 (± 4.14), *n* = 39	< 0.000
	Female	76.43 (± 5.72), *n* = 106	66.29 (± 4.04), *n* = 72	< 0.000
MCH (pg)	Male	25.53 (± 2.28), *n* = 66	21.02 (± 1.14), *n* = 39	< 0.000
	Female	24.48 (± 2.42), *n* = 106	20.45 (± 1.77), *n* = 72	< 0.000
MCHC (g/L)	Male	32.41 (± 1.38), *n* = 66	31.27 (± 1.22), *n* = 39	< 0.000
	Female	32.00 (± 1.69), *n* = 106	30.83 (± 2.21), *n* = 72	< 0.000
RDW (%)	Male	14.39 (± 2.97), *n* = 58	16.21 (± 2.11), *n* = 37	< 0.002
	Female	14.63 (± 2.07), *n* = 98	16.36 (± 2.45), *n* = 67	< 0.000
Hb A_2_ (%)	Male	2.89 (± 0.46), *n* = 65	2.41 (± 0.28), *n* = 38	< 0.000
	Female	2.82 (± 0.52), *n* = 101	2.51 (± 0.45), *n* = 71	< 0.000

Data represent mean ± SD (standard deviation). p-values less than 0.05 were considered statistically significant

Both MCV and MCH demonstrated excellent discriminatory ability between the genotypic groups in both sexes, with AUC values exceeding 0.94 (Table 7). The optimal MCV cut-off values were < 71.35 fL for males and < 70.75 fL for females. The corresponding MCH cut-off values were 22.65 pg for males and 22.25 pg for females. The sensitivity and specificity of both MCV and MCH was higher in females than in males as shown in Table 7.

**Table 7:** Summary of the cut-off values, AUC, sensitivities and specificities for all haematological parameters to differentiate between α^+^ and α⁰ thalassaemia

Parameters (unit)	Gender	Cut-off	AUC	Sensitivity	Specificity	*P*-value	95% confidence interval
RBC (x 10^12^/L)	Male	6.19	0.87	81.00	81.10	< 0.000	0.79–0.94
	Female	5.12	0.78	70.40	70.10	< 0.000	0.71–0.86
Hb (g/dL)	Male	14.45	0.65	57.90	58.3	0.014	0.54–0.76
	Female	11.85	0.62	57.00	59.10	0.080	0.54–0.71
MCV (fL)	Male	71.50	0.94	89.50	86.10	< 0.000	0.88–0.99
	Female	70.75	0.94	90.30	90.90	< 0.000	0.91–0.98
MCH (pg)	Male	22.65	0.95	89.50	88.90	< 0.000	0.90–0.99
	Female	22.25	0.94	90.30	93.90	< 0.000	0.90–0.98
MCHC (g/L)	Male	31.65	0.74	73.70	72.20	< 0.000	0.64–0.84
	Female	31.55	0.68	67.70	63.60	< 0.000	0.60–0.77
RDW (%)	Male	14.85	0.78	75.90	75.70	< 0.000	0.68–0.88
	Female	15.25	0.76	70.40	70.10	< 0.000	0.69–0.81
Hb A_2_ (%)	Male	2.55	0.82	75.40	72.20	< 0.000	0.73–0.91
	Female	2.55	0.69	67.70	65.20	< 0.000	0.61–0.77

The number of samples for α^+^ and α⁰ thalassaemia exceeded the minimum calculated sample size (25 per group)

## Discussion

The -α³·⁷ deletion is the most prevalent form of α⁺ thalassaemia across various populations, particularly in malaria-endemic regions [19]. In our study, 82.1% (*n* = 155) of α⁺ thalassaemia cases, including both − α/αα and − α/−α genotypes, carried the − α³·⁷ deletion, with a predominance among Malays (*n* = 124, 80.0%) (Table 3), consistent with findings from other local studies [5–8]. We also discovered that carriers of the --^SEA^ deletion are more common in Malays (*n* = 43, 60.6%) than in Chinese (*n* = 22, 31.0%), contradicting previous studies that identified it as the most common genotype in Chinese [5–8]. Our results may be influenced by the smaller sample size compared to the previous study [5–8], as our center currently focuses on testing complex cases and serves as a secondary molecular laboratory for further testing of α thalassaemia, due to limitations in the techniques employed by primary molecular laboratories.

We identified 40 cases involving rare deletions, including --^GB^, --^AW^, and -α^2.4^ (Table 2). The --^GB^ deletion was previously reported in individuals of Arab, Indonesian, in individuals of unknown ethnicity living in Australia, and in a Dutch patient [13, 20]. More recently, this deletion was identified in 29 Malay individuals in our study [16]. The --^AW^ deletion, initially described in a Dutch family, was discovered in six Malay individuals, while the -α^2.4^ deletion, previously found in a Chinese family in China, was identified in one Chinese individual in this study [14, 15]. The --^GB^ and --^AW^ deletions, which are 16.7 kb and 8.2 kb, respectively, remove both *HBA1* and *HBA2* genes and result in α⁰ Thalassaemia, whereas the -α^2.4^ deletion removes only the *HBA1* gene, resulting in α^+^ Thalassaemia. The use of MLPA in our center, which detects any deletions within the α-globin gene cluster, contributed significantly to these findings, as seen in another study [20]. This technique could be a valuable first-line method for analyzing suspected α thalassaemia cases.

Our study demonstrated that two parameters are strongly affected by the number of α-globin genes deleted: MCV and MCH. The strongest correlation was observed in MCH (r^2^ = 0.77), followed by MCV (r^2^ = 0.73). The more α-globin genes deleted, the lower the values of MCH and MCV. The RDW values are moderately affected at an opposite trend (r^2^ = −0.54). The high RDW in deletional Hb H disease may be attributed to increased reticulocytes, which are larger in size compared to normal red blood cells. This is exacerbated by an imbalance in the α/β-globin chain ratio in the disease, leading to precipitation of free β-globin chain tetramers in erythroid precursors, thereby causing ineffective erythropoiesis. Both of these conditions ultimately cause anisocytosis [4, 6].

In line with these findings, presumptive codes adapted from the MTDC were applied in Hb analysis reporting to indicate likely genotypes based on haematological patterns. Multiple codes were, however, assigned to α⁺ thalassaemia silent carriers, reflecting their heterogeneous haematological profiles. This variability underscores the limitations of relying solely on haematological indices for genotype discrimination. Additionally, conditions such as iron deficiency anaemia or overlapping haematological disorders can mimic thalassaemia traits, potentially leading to misclassification. Identifying α⁺ thalassaemia silent carriers is generally less critical, as couples with this genotype have only a 25% chance of producing offspring with an α⁺ thalassaemia carrier, which typically has minimal clinical impact. Screening becomes more important, however, if their partner exhibits microcytosis, which may indicate an α⁰ thalassaemia carrier and warrants molecular testing [4, 9].

The identification of individuals with deletional Hb H disease is also another of great clinical importance due to the reproductive risks involved, as they may have children with deletional Hb H or Hb Bart’s disease if their partner has α^+^ thalassaemia or α⁰ Thalassaemia, respectively. Deletional Hb H disease is typically straightforward to diagnose based on clinical symptoms and Hb analysis, as seen in this study, 81.0% (*n* = 17) of such cases were correctly assigned code A1.

On the other hand, α⁰ thalassaemia carrier is more challenging to detect, as its haematological features closely resemble those of α^+^ thalassaemia carrier, making DNA analysis the only accurate way for differentiation [1, 4, 8, 9]. Nevertheless, confirming all potential cases is impractical due to the prevalence of the α^+^ form, which typically lacks clinical significance. In addition, this approach is not cost-effective and may cause unnecessary anxiety among carriers [6, 9].

In this study, nearly all α⁺ and α⁰ thalassaemia carriers were assigned the D13 code, with 91.7% (*n* = 11) and 92.8% (*n* = 103) of cases, respectively. In contrast, only 19.9% (*n* = 32) of α⁺ thalassaemia silent carriers received the D13 code (Table 5). These findings indicate that while the MCH cut-off of 25 pg used in the MTDC effectively identifies α⁰ thalassaemia carriers, it does not distinguish them from α⁺ thalassaemia carriers, highlighting the need for DNA analysis for accurate diagnosis [6, 8, 21].

This study also showed that none of the α⁰ thalassaemia carrier cases were coded as D16, indicating that the presumptive screening effectively avoided misclassification of these cases. Since the D16 code generally does not prompt DNA analysis, its absence in this group is important to ensure true α⁰ carriers are not overlooked. The D16 code is more appropriately associated with α^+^ thalassaemia cases, especially in silent carriers and, to a lesser extent, in α^+^ thalassaemia carriers, as a means of reducing unnecessary DNA testing. This pattern was correctly reflected in our study, where none of the α^+^ thalassaemia carrier cases were coded as D16; though this finding may be confounded by the small sample size (*n* = 12).

Our study demonstrated that, in both genders, all haematological parameters of α⁰ thalassaemia carriers, except Hb level, were significantly different from those of the α⁺ groups (-α/αα and -α/-α) (Table 6). Cut-off values of < 71.35 fl. for MCV and < 22.65 pg for MCH effectively identified male α⁰ thalassaemia carriers, while < 70.75 fl. for MCV and < 22.25 pg for MCH were optimal for females (Table 7). Both parameters showed comparable discriminatory efficiency in both genders (AUC 0.94–0.95). Previous studies have proposed lower MCH cut-offs, such as 23.5 pg by Idris et al. (2020) [8] and 21.5 pg by Velasco-Rodríguez et al. (2017) [1], but these were not stratified by gender. Our gender-specific analysis indicates that slightly lower cut-offs in females improve discrimination between α⁰ and α⁺ thalassaemia carriers in our population. These findings support using MCH as the preferred screening parameter due to its stability compared to MCV [9, 18, 22]. By adjusting the MCH cut-off for D13 and expanding the MCH range for D16, more α⁺ thalassaemia cases could be excluded from further DNA testing while reliably detecting α⁰ thalassaemia carriers, providing evidence-based reference points to potentially revise the current screening cut-off (MCH < 25 pg).

An important limitation of this study is the uneven distribution of sample sizes, with only 12 cases in the α⁺ thalassaemia carrier (-α/-α) group and 21 cases in the deletional Hb H disease (--/-α) group. This reflects the referral nature of the IMR, a secondary center for molecular diagnosis in Malaysia, which receives complex cases from primary laboratories. Consequently, many study cases were derived from parental samples of complicated index cases, contributing to the small numbers in some genotype groups and limiting the generalizability of the findings. Another limitation is the unknown iron status in most cases, which is relevant given the high prevalence of iron deficiency in our population [23]. Because MCH is influenced by both iron deficiency anaemia and α⁰ thalassaemia [24], concurrent iron deficiency in some individuals may have affected the haematological parameters and the interpretation of cut-offs. In addition, the non-deletional α-thalassaemia cases were not included in our analysis. These variants, including Hb Constant Spring, represent a distinct category with different haematological profiles and cut-off values [6, 7], and were therefore excluded to maintain consistency in the evaluation of deletional α-thalassaemia. Their absence may limit the applicability of our findings to all α-thalassaemia genotypes. Finally, not all of the deletional forms of α thalassaemia were evaluated, as 37 cases with uncharacterized deletions were excluded from this study.

## Conclusions

Definitive diagnosis of α thalassaemia can only be achieved through molecular studies; however, haematological parameters serve as valuable predictive markers for estimating the number of α-globin gene deletions. This study demonstrated that using a lower MCH cut-off than the current threshold employed in the MTDC can more effectively detect α⁰ thalassaemia carriers, potentially reducing unnecessary molecular testing and overall laboratory workload. Based on our findings, we propose that molecular testing be prioritized for couples in which either partner has an MCH value below the revised cut-off, to avoid missing individuals with two gene deletions on one allele (α⁰ thalassaemia carrier, --/αα). This approach may contribute to further reducing the incidence of deletional Hb H disease and Hb Bart’s hydrops foetalis. Nonetheless, larger-scale studies incorporating a wider range of common α thalassaemia mutations in the region are warranted to validate the utility of this adjusted MCH cut-off for guiding molecular testing decisions.

## Data Availability

The data for this study is held at the Institute for Medical Research, Ministry of Health, Malaysia. Restrictions apply to the availability of these data, which were used under license for the current study and are not publicly available. However, data may be made available by the corresponding author upon reasonable request and with prior approval from the Ministry of Health Malaysia.
